# Transferrin Receptor Protein 1 Is an Entry Factor for Rabies Virus

**DOI:** 10.1128/jvi.01612-22

**Published:** 2023-02-13

**Authors:** Xinxin Wang, Zhiyuan Wen, Huizhen Cao, Jie Luo, Lei Shuai, Chong Wang, Jinying Ge, Xijun Wang, Zhigao Bu, Jinliang Wang

**Affiliations:** a State Key Laboratory of Veterinary Biotechnology, Harbin Veterinary Research Institute, Chinese Academy of Agricultural Sciences, Harbin, People’s Republic of China; The Peter Doherty Institute for Infection and Immunity

**Keywords:** rabies virus, TfR1, entry factor, endocytosis

## Abstract

Rabies virus (RABV) is a prototypical neurotropic virus that causes rabies in human and animals with an almost 100% mortality rate. Once RABV enters the central nervous system, no treatment is proven to prevent death. RABV glycoprotein (G) interacts with cell surface receptors and then enters cells via clathrin-mediated endocytosis (CME); however, the key host factors involved remain largely unknown. Here, we identified transferrin receptor 1 (TfR1), a classic receptor that undergoes CME, as an entry factor for RABV. TfR1 interacts with RABV G and is involved in the endocytosis of RABV. An antibody against TfR1 or the TfR1 ectodomain soluble protein significantly blocked RABV infection in HEK293 cells, N2a cells, and mouse primary neuronal cells. We further found that the endocytosis of TfR1 is coupled with the endocytosis of RABV and that TfR1 and RABV are transported to early and late endosomes. Our results suggest that RABV hijacks the transport pathway of TfR1 for entry, thereby deepening our understanding of the entry mechanism of RABV.

**IMPORTANCE** For most viruses, cell entry involves engagement with many distinct plasma membrane components, each of which is essential. After binding to its specific receptor(s), rabies virus (RABV) enters host cells through the process of clathrin-mediated endocytosis. However, whether the receptor-dependent clathrin-mediated endocytosis of RABV requires other plasma membrane components remain largely unknown. Here, we demonstrate that transferrin receptor 1 (TfR1) is a functional entry factor for RABV infection. The endocytosis of RABV is coupled with the endocytosis of TfR1. Our results indicate that RABV hijacks the transport pathway of TfR1 for entry, which deepens our understanding of the entry mechanism of RABV.

## INTRODUCTION

Rabies, caused by rabies virus (RABV), is a devastating infectious disease that has a long history and continues to affect 40,000 to 70,000 individuals globally each year (1). Humans are usually infected through contact with RABV-infected animals (1). Once the virus invades the central nervous system, no therapy has been proven to prevent death, and the mortality rate is almost 100%.

RABV is a prototypical neurotropic virus and belongs to the genus *Lyssavirus* of the family *Rhabdoviridae* (1). The RABV genome consists of five genes that respectively encode five proteins: nucleoprotein, phosphoprotein, matrix protein, glycoprotein (G), and the RNA-dependent RNA polymerase protein. RABV G is the only protein exposed on the surface of the viral envelope (2) and is responsible for receptor binding and viral entry (3, 4). After binding to its host receptor(s), RABV enters host cells through receptor-dependent clathrin-mediated endocytosis (CME) (4–6).

Virus entry is a dynamic and complex process. The virus-receptor interaction is essential for virus entry, which plays a key regulatory role in viral host range, tissue tropism, and viral pathogenesis (7, 8). Previous studies have indicated that RABV may use multiple receptors for infection (9), and at least four proteins—acetylcholine receptor subunit alpha (10), neural cell adhesion molecule (11), low-affinity nerve-growth factor receptor (12), and metabotropic glutamate receptor subtype 2 (mGluR2) (13)—have been identified as potential receptors for RABV infection *in vitro*. In addition to viral receptors, increasing evidence indicates that the entry of many viruses involves engagement with distinct plasma membrane components as entry factors, each of which is important (8). Recent studies have reported that integrin β1 (14) and AP-2-associated protein kinase 1 (15, 16) play roles in RABV entry; however, the key host factors involved in the endocytosis of RABV remain largely unknown.

In our previous study, we used a global RNAi strategy to screen potential host factors required for RABV infection in HEK293 cells. We found that human transferrin receptor 1 (TfR1), a type II transmembrane protein that undergoes classic CME (17), is required for RABV infection. TfR1 is ubiquitously expressed in almost all tissues while transferrin receptor 2 (TfR2) is mainly expressed in liver (18). Both TfR1 and TfR2 are involved in the cellular transport of iron into cells through the binding of iron-loaded transferrin (Tf) (17–19). Treatment with ferric ammonium citrate (FAC) downregulates the expression of TfR1 in cells (20, 21). In this study, we identified TfR1 as an entry factor for RABV. We found that TfR1 interacts with RABV G directly and that the endocytosis of TfR1 is required for the endocytosis of RABV. RABV and TfR1 were internalized into cells and transported to early and late endosomes together. Our study suggests that TfR1 is a key host factor involved in the endocytosis of RABV, and provides a potential target for antiviral development against RABV infection.

## RESULTS

### TfR1 is required for RABV infection.

**FIG 1:**
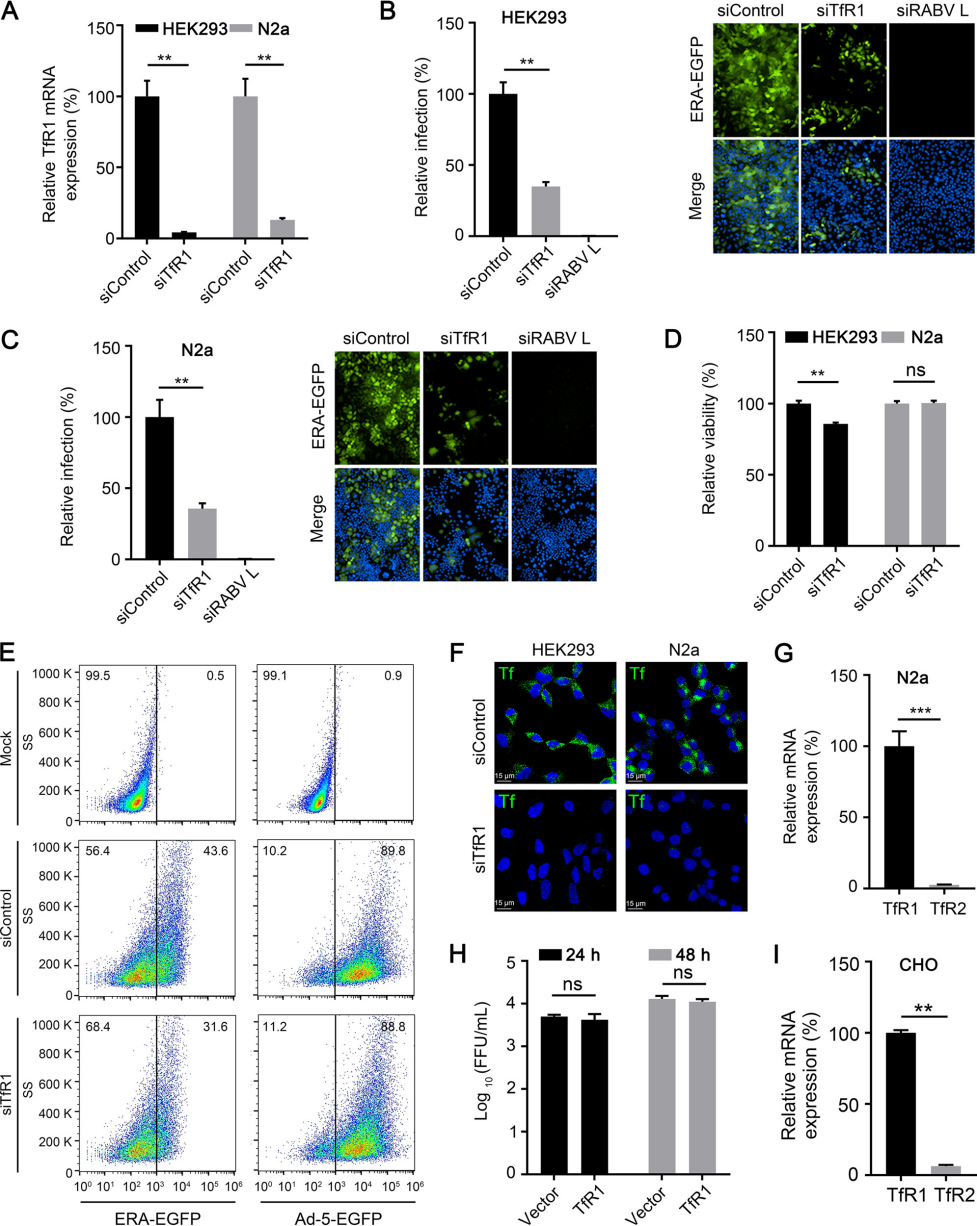
Silencing TfR1 expression inhibits RABV infection. (A) The TfR1 mRNA level in the indicating siRNA-transfected HEK293 cells or N2a cells was measured by qPCR. siTfR1, siRNAs specific for TfR1 mRNA; siControl, scrambled RNA. (B and C) TfR1-silenced HEK293 cells (B) or N2a cells (C) were infected with ERA-EGFP for 48 h at 37°C. The cell nuclei were stained (blue). The percentages of infected cells and representative images are shown. (D) The viability of TfR1-silenced HEK293 cells or N2a cells at 60 h posttransfection was determined by using the Cell Titer Glo kit. (E) TfR1-silenced HEK293 cells were infected with ERA-EGFP or Ad-5-EGFP for 24 h at 37°C. The percentage of infected cells was detected by using flow cytometry. (F) siControl- or siTfR1-transfected HEK293 cells or N2a cells were incubated with fluorescent Tf for 30 min at 37°C. Then, fluorescent Tf was detected by microscopy. Representative images are shown. (G) The TfR1 mRNA and TfR2 mRNA levels in N2a cells were measured by qPCR. (H) Human TfR1-overexpressed CHO cells were infected with ERA-EGFP. At 24 and 48 h postinfection, the supernatants were harvested to determine virus titers. (I) The TfR1 mRNA and TfR2 mRNA level in CHO cells were measured by qPCR. The data shown are means ± the SD of three independent experiments or replicates. The two-tailed unpaired Student *t* test was used for the statistical analysis. ns, not significant; **, *P* < 0.01; ***, *P* < 0.001.

Previously, we performed a high-throughput small interfering RNA (siRNA) screen to identify host factors required for RABV infection and found that mGluR2 is an entry receptor of RABV (13). Subsequent analyses revealed that knockdown of TfR1 expression significantly inhibited the replication of ERA-EGFP, a recombinant RABV ERA strain that expresses enhanced green florescent protein. To confirm that TfR1 is required for RABV infection, we knocked down TfR1 expression by transfecting HEK293 cells and N2a cells (a mouse neuroblastoma cell line) with specific siRNA to TfR1 mRNA. Real-time quantitative PCR (qPCR) showed that compared to siControl-transfected cells, the expression of TfR1 mRNA in siTfR1-transfected cells was significantly reduced in both cell lines at 24 h posttransfection (Fig. 1A). At 60 h posttransfection, HEK293 cells and N2a cells were each infected with ERA-EGFP. The infected cells were quantified by use of microscopy at 48 h postinfection, and the results showed that knockdown of TfR1 strongly inhibited the replication of ERA-EGFP in both cell lines (Fig. 1B and C). We also examined the viability of TfR1-silenced HEK293 cells and N2a cells at 60 h posttransfection and found that the cell viability had slightly and significantly decreased in TfR1-silenced HEK293 cells but not significant in N2a cell viability (Fig. 1D). We then used recombinant adenovirus 5 expressing enhanced green fluorescent protein (Ad-5-EGFP) as a control to exclude the possibility of a cytotoxic effect of silencing TfR1 on RABV infection in HEK293 cells. At 60 h posttransfection with siTfR1, cells were infected with Ad-5-EGFP, and infected cells were quantified by use of flow cytometry at 24 h postinfection. The results showed that knockdown of TfR1 had no effect on Ad-5-EGFP infection in HEK293 cells, which indicates that the slight cytotoxicity induced by silencing TfR1 had no effect on RABV infection (Fig. 1E). These results suggest that TfR1 is an important host factor for RABV infection.

Since TfR1 and TfR2 function similarly to mediate the endocytosis of Tf (18), we next performed Tf uptake and qPCR assays to detect the expression of TfR1 and TfR2 in cells. Compared to siControl-transfected cells, the uptake of Tf in siTfR1-transfected HEK293 cells and N2a cells was significantly decreased at 60 h posttransfection (Fig. 1F). While both TfR1 mRNA and TfR2 mRNA were detected in N2a cells, the expression of TfR2 mRNA was much less than that of TfR1 mRNA (Fig. 1G). In addition, the data from the Human Protein Alts database showed that TfR2 mRNA was not detected in HEK293 cells (22) (https://www.proteinatlas.org/ENSG00000106327-TFR2/cell+line). It had been reported that overexpression of TfR1 or TfR2 significantly increased the uptake of Tf in CHO cells, which indicates that CHO cells were negative or low in expression of TfR1 or TfR2 (19). We further performed RABV infection assay in human TfR1-overexpressed CHO cells. The results showed that overexpression of TfR1 had no effect on RABV infection (Fig. 1H). qPCR assays revealed that while both TfR1 mRNA and TfR2 mRNA were detected in CHO cells, the expression of TfR2 mRNA was much less than that of TfR1 mRNA (Fig. 1I). Therefore, the endocytosis of RABV is mainly mediated by TfR1 in cells.

### Ferric ammonium citrate inhibits RABV infection.

**FIG 2:**
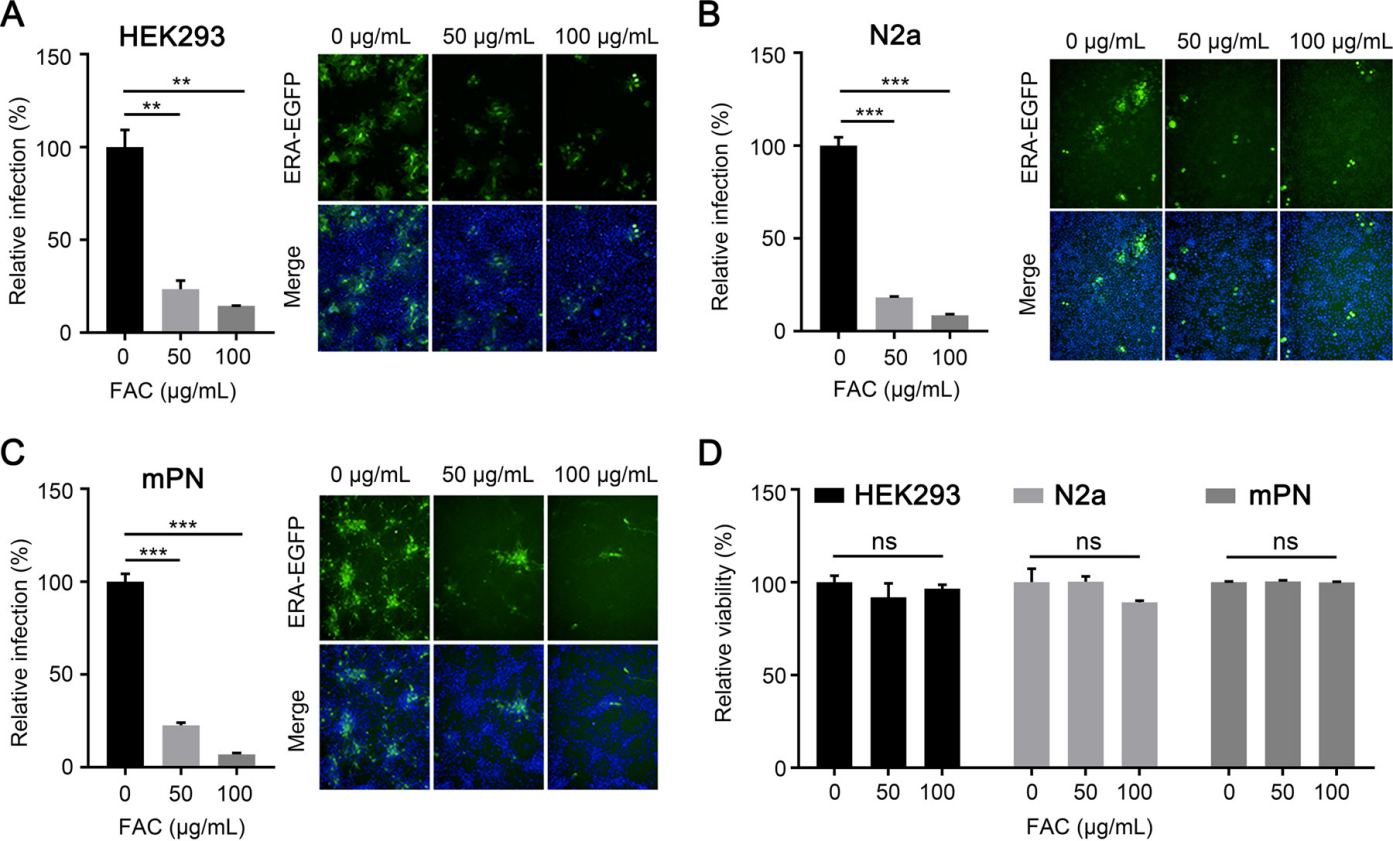
FAC inhibits RABV infection. HEK293 cells (A), N2a cells (B), or mouse primary neuron (mPN) cells (C) were treated with FAC at the indicated concentrations for 1 h and then infected with ERA-EGFP for 48 h at 37°C. The cell nuclei were stained (blue). The percentages of infected cells and representative images are shown. (D) HEK293 cells, N2a cells, or mPN cells were treated with FAC at different concentrations for 48 h at 37°C, and then cell viability was determined by using the Cell Titer Glo kit. The data shown are means ± the SD of three independent experiments or replicates. A two-tailed unpaired Student *t* test was used for the statistical analysis. ns, not significant; **, *P* < 0.01; ***, *P* < 0.001.

To confirm above results, we performed an inhibitor assay in HEK293 cells and N2a cells by treating these cells with the TfR1 inhibitor FAC. FAC downregulates TfR1 expression in cells (20, 21). The results showed that FAC significantly inhibited the replication of RABV in a dose-dependent manner at 48 h postinfection in HEK293 cells and N2a cells (Fig. 2A and B). We then performed the inhibitor assay in mouse primary neuronal (mPN) cells, and found that FAC significantly decreased the replication of RABV in a dose-dependent manner (Fig. 2C). We also tested the cytotoxicity of FAC in HEK293 cells, N2a cells, and mPN cells and found that the concentrations of FAC used showed low toxicity to all three cell lines (Fig. 2D). These results confirm that TfR1 is an important host factor for RABV infection.

### TfR1 directly interacts with RABV G.

**FIG 3:**
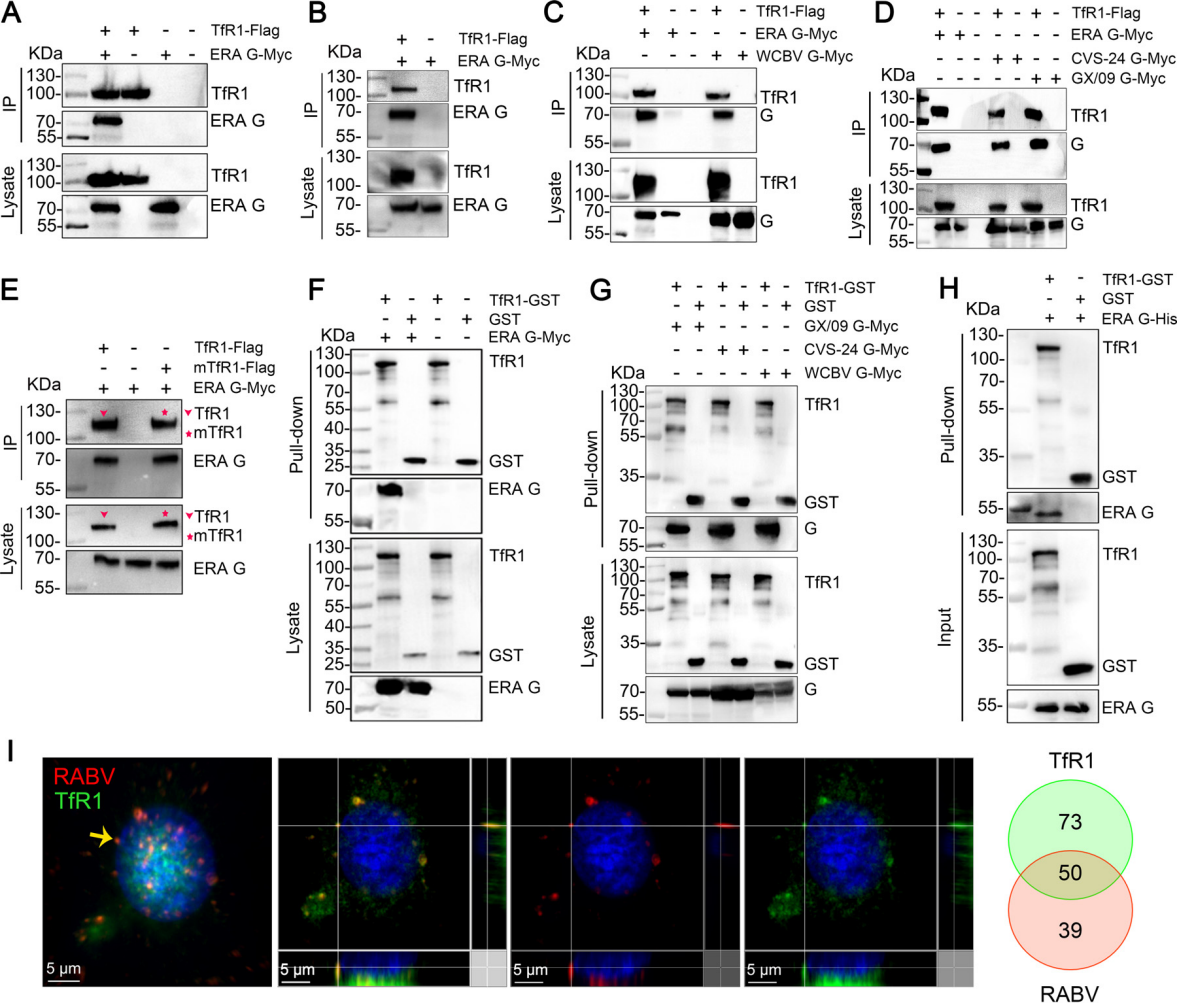
TfR1 directly interacts with RABV G. HEK293 cells were cotransfected with TfR1-Flag and ERA G-Myc (A and B), WCBV G-Myc (C), or CVS-24 G-Myc or GX/09 G-Myc (D). Then, the cells were solubilized by NP-40 lysis buffer (A, C, and D) or RIPA lysis buffer (B) and subjected to immunoprecipitation (IP) using anti-Flag agarose beads. (E) HEK293 cells were cotransfected with TfR1-Flag or mouse TfR1-Flag (mTfR1-Flag) and ERA G-Myc, and then the cells were lysed by NP-40 lysis buffer, and then subjected to IP by using anti-Flag agarose beads. (F and G) Purified TfR1-GST protein was pooled with the lysate from ERA G-Myc-transfected (F) or CVS-24 G-Myc-, GX/09 G-Myc-, or WCBV G-Myc-transfected (G) HEK293 cells and then pulled down using anti-GST beads. (H) TfR1-GST was pooled with ERA G-His and then pulled down using anti-GST beads. The GST protein was used as the negative control. (I) Multiplex immunofluorescence was performed in N2a cells. Colocalization of TfR1 (green) and ERA-N-mCherry (red) was observed (left) and quantified (right). The yellow arrow indicates the representative colocalization of RABV and TfR1, shown in three dimensions. The Venn diagram represents the total amount of colocalization of RABV (red) with TfR1 (green). The data shown are representative of three independent experiments.

TfR1 is ubiquitously expressed in almost all tissues (23). We hypothesized that TfR1 may serve as an entry factor for RABV infection; a direct interaction between TfR1 and RABV G would strongly support this hypothesis. We therefore performed a coimmunoprecipitation assay using Flag-tagged TfR1 protein (TfR1-Flag) and Myc-tagged RABV G derived from the cell-adapted strain ERA, the mouse-adapted strain CVS-24, the street virus GX/09, or another member of the *Rhabdoviridae* family West Caucasian bat virus (WCBV) in plasmid-transfected HEK293 cells. We found that TfR1 interacted with ERA G (Fig. 3A and B) and WCBV G, CVS-24 G, and GX/09 G (Fig. 3C and D). Given that TfR1 also plays an important role in RABV infection of N2a cells, we performed coimmunoprecipitation assay to detect whether mouse transferrin receptor 1 (mTfR1) interacts with RABV G. The result showed that mTfR1 interacts with ERA G (Fig. 3E).

We then performed pulldown assays to determine whether the ectodomain of TfR1 is responsible for the interaction between TfR1 and RABV G. The purified recombinant glutathione *S*-transferase-tagged TfR1 protein ectodomain (TfR1-GST) was pooled with the lysate from HEK293 cells that had been transfected with ERA G-Myc, CVS-24 G-Myc, GX/09 G-Myc, or WCBV G-Myc plasmids. We found that the ectodomain of TfR1 successfully pulled down ERA G, CVS-24 G, GX/09 G, and WCBV G (Fig. 3F and G). We then performed the pulldown assay using TfR1-GST and purified His-tagged ERA G ectodomain (ERA G-His) protein to determine whether the ectodomain of TfR1 and the ectodomain of RABV G interact directly. The result showed that TfR1-GST pulled down ERA G-His (Fig. 3H). These results indicate that the ectodomains of TfR1 and RABV G interact directly.

Next, we examined the localization of TfR1 and RABV particles in infected cells, as previously described (13). N2a cells were infected with ERA-N-mCherry, a recombinant ERA expressing an additional open reading frame of N protein fused with mCherry. At 10 min postinfection, immunofluorescence with tyramide signal amplification (TSA) was performed to detect TfR1 and ERA-N-mCherry. The results showed that TfR1 and RABV particles colocalized in infected cells (Fig. 3I). These results suggest that TfR1 directly interacts with RABV G.

### TfR1 antibody blocks RABV infection.

**FIG 4:**
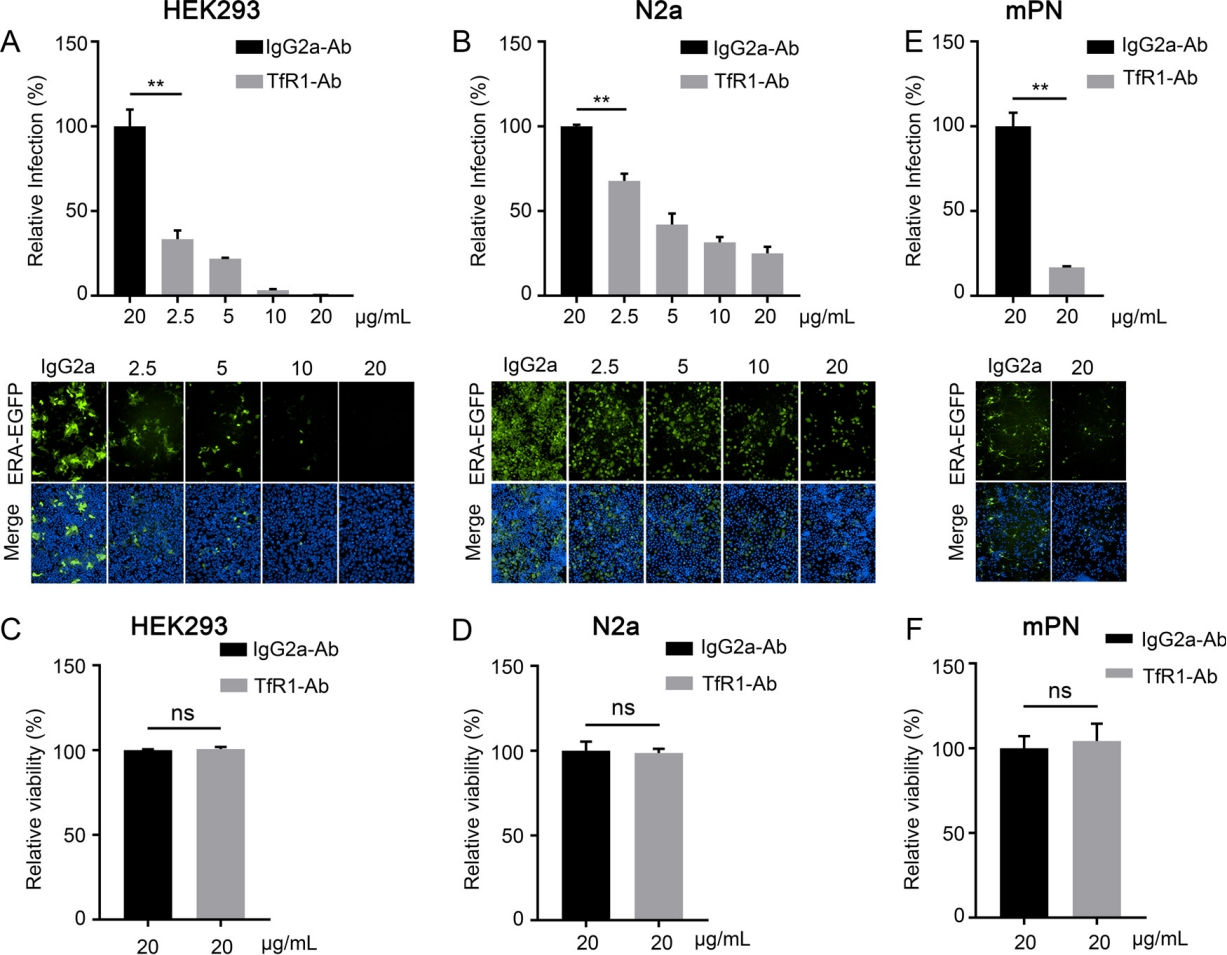
An antibody against TfR1 blocks RABV infection. HEK293 cells (A), N2a cells (B), or mPN cells (E) were treated with TfR1 antibody at different concentrations or IgG2a (20 μg/mL) for 1 h at 4°C and then infected with ERA-EGFP. The cell nuclei were stained (blue). The percentages of infected cells and representative images are shown. HEK293 cells (C), N2a cells (D), or mPN cells (F) were treated with TfR1 antibody or IgG2a (20 μg/mL) for 48 h. Cell viability was determined by using a Cell Titer Glo kit. The data shown are means ± the SD of three independent experiments. The two-tailed unpaired Student *t* test was used for the statistical analysis. ns, not significant; **, *P* < 0.01.

To investigate whether TfR1 serves as an entry factor for RABV infection, we tested whether an antibody against the ectodomain of TfR1 could block RABV infection *in vitro*. HEK293 cells and N2a cells were treated with the TfR1 antibody at different concentrations for 1 h at 4°C and then incubated with ERA-EGFP. At 48 h postinfection, infected cells were quantified by use of microscopy. The results showed that the TfR1 antibody efficiently inhibited RABV infection of HEK293 cells and N2a cells (Fig. 4A and B) and that cell viability was unaffected at the concentrations tested (Fig. 4C and D). We also performed the test in mPN cells and confirmed that the TfR1 antibody significantly decreased RABV infection of cells and that it was not cytotoxic at the concentrations used (Fig. 4E and F).

### The TfR1 ectodomain soluble protein inhibits RABV infection.

**FIG 5:**
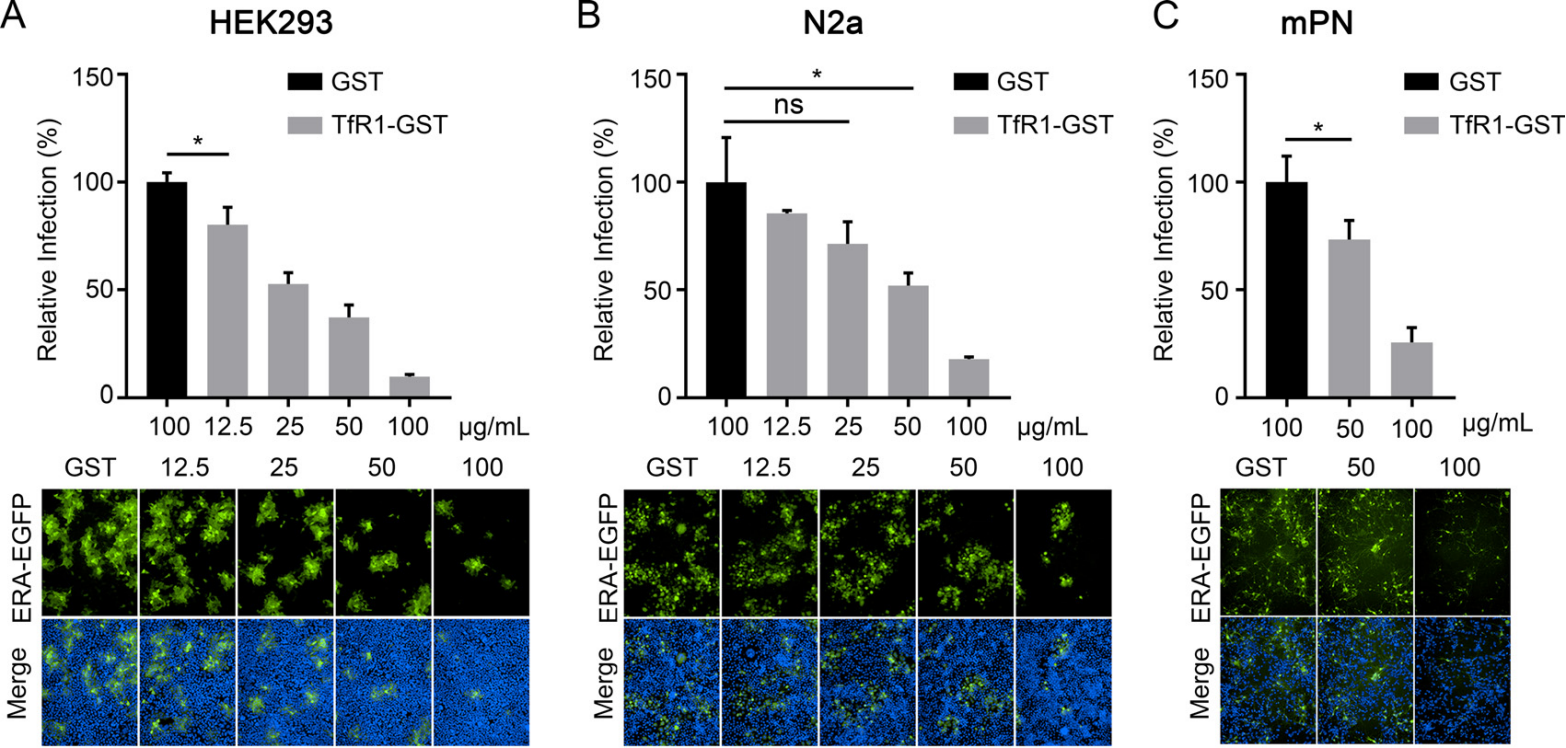
The TfR1 ectodomain soluble protein (TfR1-GST) inhibits RABV infection. ERA-EGFP was pooled with different concentrations of TfR1-GST or GST (100 μg/mL), and then HEK293 cells (A), N2a cells (B), and mPN cells (C) were infected with the different mixtures, respectively. The GST protein was used as the negative control. The cell nuclei were stained (blue). The percentages of infected cells and representative images are shown. The data shown represent, or are from, three independent experiments or replicates (means ± the SD). The two-tailed unpaired Student *t* test was used for the statistical analysis. ns, not significant; *, *P* < 0.05.

If RABV uses TfR1 as an entry factor, the soluble TfR1 protein should inhibit RABV infection. We therefore performed neutralization assays *in vitro* by using ERA-EGFP and TfR1-GST. ERA-EGFP was pooled with different concentrations of TfR1-GST for 1 h at 4°C, and then the cells were incubated with the mixtures for 1 h at 37°C. At 48 h postinfection, the infected cells were quantified by use of microscopy. We found that TfR1-GST inhibited RABV infection in a dose-dependent manner in HEK293 cells, N2a cells, and mPN cells (Fig. 5). The results suggest that TfR1 is an important entry factor for RABV infection *in vitro*.

### TfR1 is required for the endocytosis of RABV.

**FIG 6:**
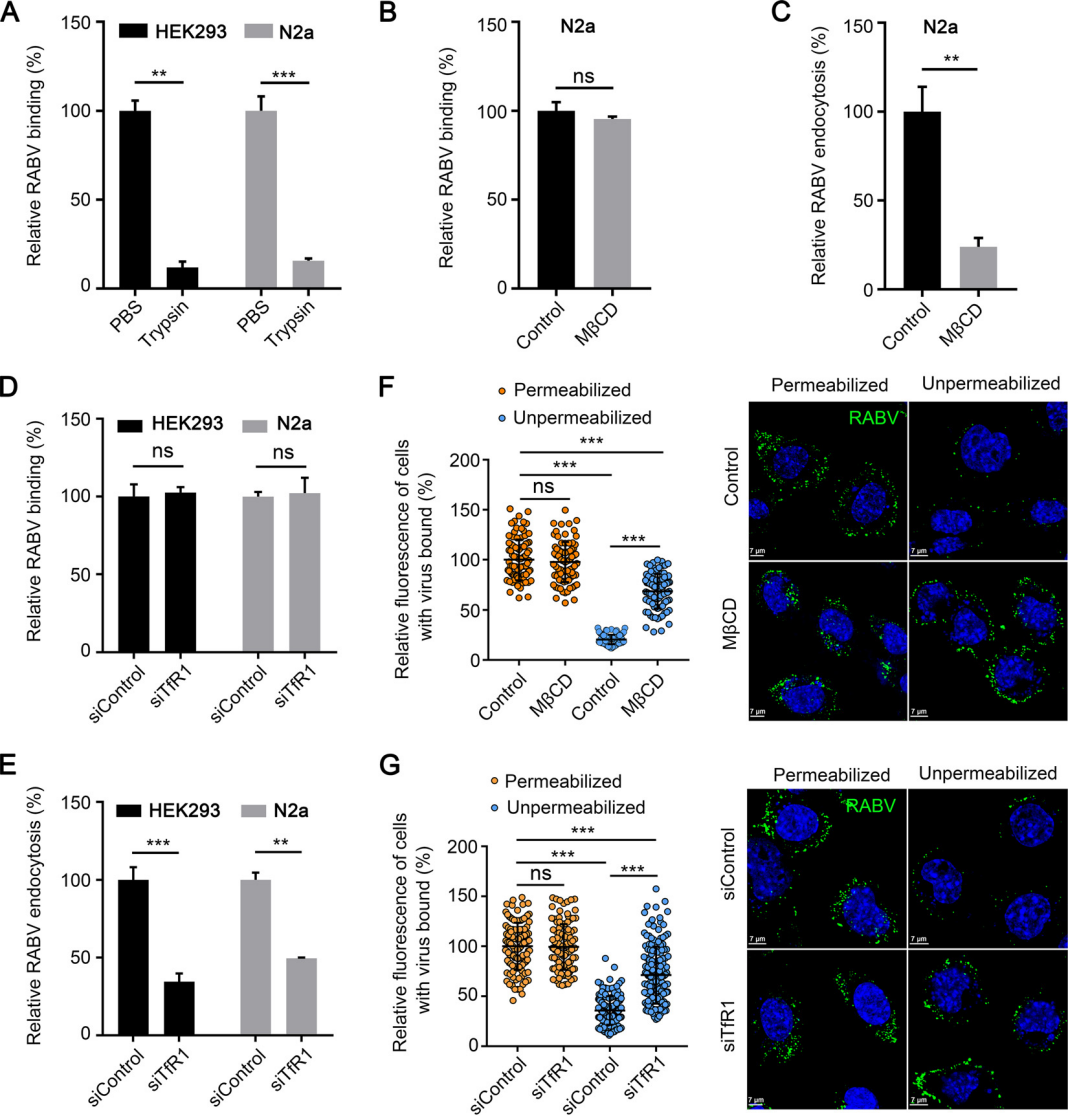
TfR1 is required for the endocytosis of RABV. (A) HEK293 cells or N2a cells were incubated with RABV for 1 h at 4°C and then washed with trypsin and lysed for qPCR to detect RABV bound to the cell surface. RABV binding (B) and entry (C) assays were performed with MβCD-treated (10 mM) N2a cells. Viral binding or entry was quantified by normalization to the respective control cells. RABV binding (D) and entry (E) assays were performed with TfR1-silenced HEK293 cells and N2a cells, respectively. Viral binding or entry was quantified by normalization to the respective control cells. (F) MβCD-treated N2a cells were processed as described for panel E, except that they were not treated with trypsin. RABV was stained with an antibody against RABV G (green). Cell nuclei were stained with Hoechst 33342. Representative images are shown. The fluorescence signal of RABV particles was quantified by using ZEN software. The relative fluorescence of cell-bound RABV under permeabilized or unpermeabilized conditions was quantified by normalization to the control cells under permeabilized conditions. The circles represent individual data points. At least 80 cells per sample were quantified. (G) TfR1-silenced N2a cells were processed as described for panel F. In panels A to E, the data shown are means ± the SD of three independent experiments or replicates. In panels F and G, the data represent the sum of three independent experiments. A two-tailed unpaired Student *t* test was used for the statistical analysis. ns, not significant; **, *P* < 0.01; ***, *P* < 0.001.

We next tested which stage of RABV entry involves TfR1. We performed RNAi assays to determine whether knocking down TfR1 expression affects the binding or endocytosis of RABV. TfR1-silenced HEK293 cells, N2a cells, and control cells were incubated with ERA-EGFP at 4°C for 1 h and washed to remove unbound virus. The cells were then shifted to 37°C for 2.5 h to allow the internalization of bound viruses. The cells were washed with normal phosphate-buffered saline (PBS) or trypsin to remove cell surface-bound RABV (Fig. 6A). As a control, N2a cells were treated with methyl β-cyclodextrin (MβCD) at 37°C for 1 h and then processed as described above. MβCD sequesters cholesterol from the cell membrane, thus inhibiting CME (24, 25). The washed cells were lysed for qPCR detection of RABV that was bound to the cell surface or had entered the cells. MβCD treatment significantly inhibited the endocytosis of ERA-EGFP but not binding in N2a cells (Fig. 6B and C). We also found that TfR1 silencing had no effect on RABV binding to HEK293 cells or N2a cells (Fig. 6D) but significantly decreased the endocytosis of RABV in both cell types (Fig. 6E). These results indicate that TfR1 is important for the endocytosis of RABV.

To confirm these findings, we performed a microscopy-based assay to monitor the endocytosis of RABV as previously described (26). Briefly, TfR1-silenced N2a cells or control cells were incubated under unpermeabilized or permeabilized conditions with an antibody against the RABV G and then stained to visualize the viral particles. The fluorescence intensity of each cell was calculated. Under unpermeabilized conditions, the interaction between the antibody and the internalized viruses is blocked, so the fluorescence intensity indicates how many viral particles are unable to enter the cells, whereas that under permeabilized conditions indicates the total number of viral particles. MβCD treatment was also used as a control. The results showed that MβCD treatment significantly inhibited the endocytosis of RABV while having no effect on binding to N2a cells (Fig. 6F). The fluorescence intensity in TfR1-silenced N2a cells was significantly higher than that of control cells under unpermeabilized conditions but similar to that of control cells under permeabilized conditions (Fig. 6G). These results further demonstrate that TfR1 is required for the endocytosis of RABV.

### The endocytosis of TfR1 is required for the endocytosis of RABV.

**FIG 7:**
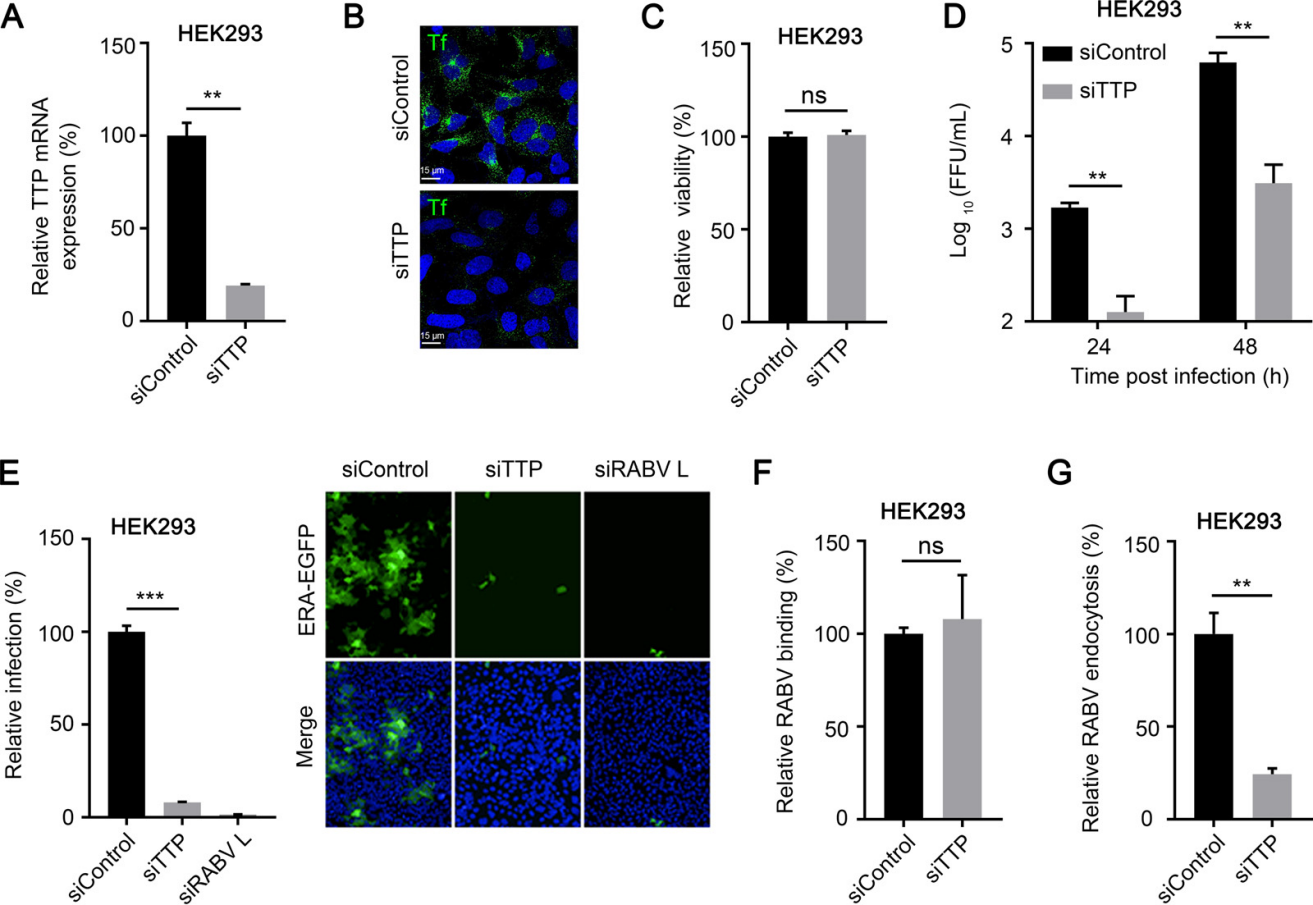
TTP is required for the endocytosis of RABV but not binding. (A) The TTP mRNA level in the indicated siRNA-transfected HEK293 cells was measured by qPCR. siTTP, siRNAs specific for TTP mRNA; siControl, scrambled RNA. (B) siControl- or siTTP-transfected HEK293 cells were incubated with fluorescent Tf for 30 min at 37°C. Then, fluorescent Tf was detected by microscopy. Representative images are shown. (C) The viability of TTP-silenced HEK293 cells at 60 h posttransfection was determined by using the Cell Titer Glo kit. (D) TTP-silenced HEK293 cells were infected with ERA-EGFP. At 24 and 48 h postinfection, the supernatants were harvested to determine virus titers. (E) TTP-silenced HEK293 cells were infected with ERA-EGFP for 48 h at 37°C. The cell nuclei were stained (blue). The percentages of infected cells and representative images are shown. RABV binding (F) and entry (G) assays were performed in TTP-silenced HEK293 cells. The data shown are means ± the SD of three independent experiments or replicates. A two-tailed unpaired Student *t* test was used for the statistical analysis. ns, not significant; **, *P* < 0.01; ***, *P* < 0.001.

Since both TfR1 and RABV enter cells through CME (5, 17), it is highly possible that RABV hijacks the endocytosis pathway of TfR1 to enter cells. A previous study showed that the endocytosis of TfR1 is specifically regulated by transferrin receptor trafficking protein (TTP) and that knockdown of TTP significantly inhibits the endocytosis of TfR1 (27). Therefore, we tested whether knockdown of TTP affects RABV infection. Our qPCR analysis confirmed that TTP mRNA expression was significantly reduced at 24 h posttransfection (Fig. 7A). An analysis of the uptake of fluorescent Tf also confirmed that cell surface expression of TTP was significantly reduced at 60 h posttransfection in HEK293 cells (Fig. 7B). The viability of TTP-silenced HEK293 cells was unaffected at 60 h posttransfection (Fig. 7C). At 60 h posttransfection, HEK293 cells were infected with ERA-EGFP, and viruses in the supernatants of the infected cells were detected by viral titration at different times postinfection. The viruses in the infected cells were quantified at 48 h postinfection. The results showed that knockdown of TTP strongly inhibited RABV infection and decreased viral titers in HEK293 cells (Fig. 7D and E).

We then performed RNAi assays to determine whether knockdown of TTP expression affects the binding or endocytosis of RABV. We found that knockdown of TTP significantly inhibited the endocytosis of RABV, while having no effect on cell binding (Fig. 7F and G), suggesting that the endocytosis of TfR1 is required for the endocytosis of RABV. These results strongly suggest that RABV hijacks the endocytic machinery of TfR1 to enter cells.

### RABV and TfR1 are internalized into cells and transported to early and late endosomes together.

**FIG 8:**
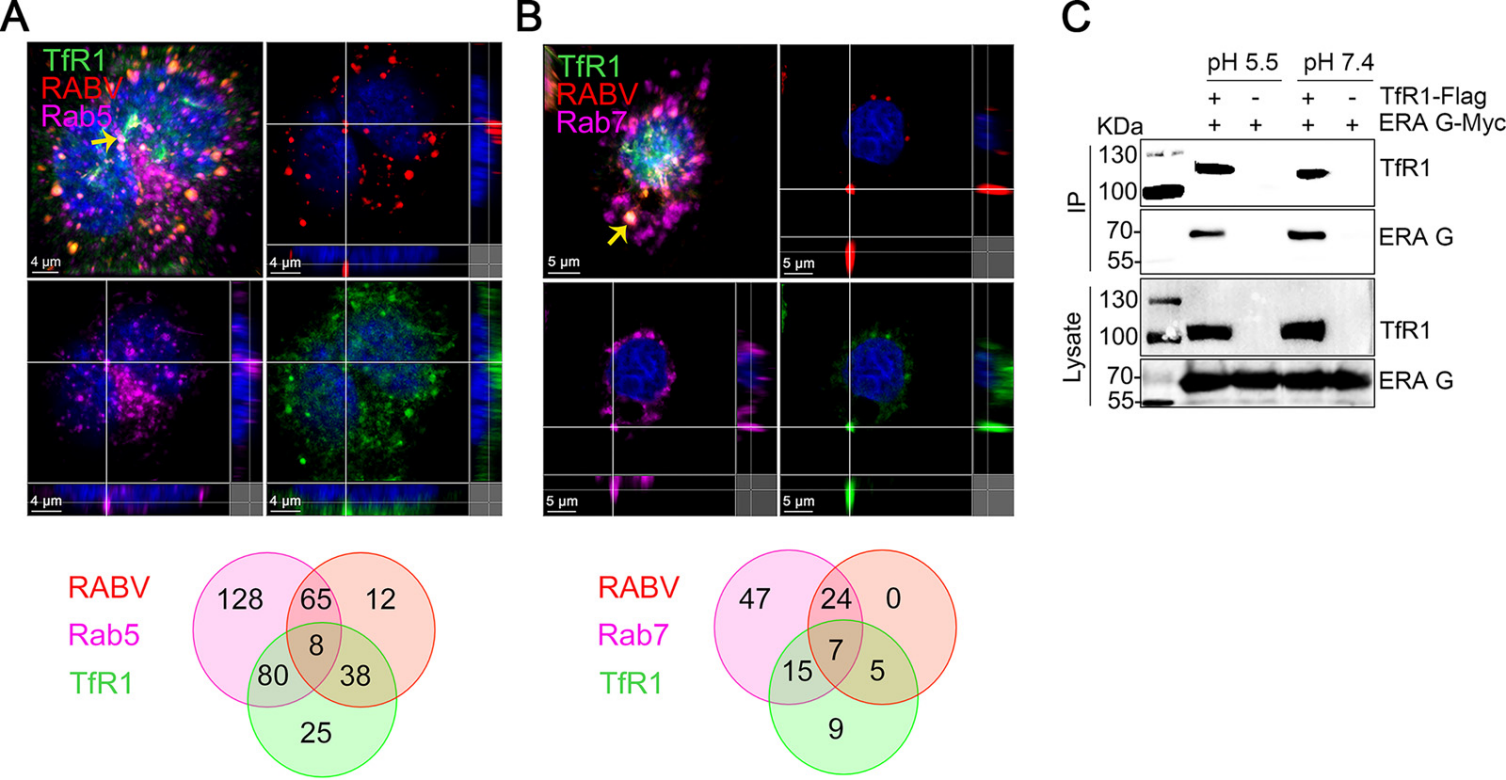
RABV and TfR1 are internalized into cells and transported together in early and late endosomes. (A and B) N2a cells were infected with ERA-N-mCherry for 20 min at 37°C, and then multiplex immunofluorescence was performed. Colocalization of TfR1 (green), ERA-N-mCherry (red), and Rab5 (purple), or Rab7 (purple) was observed (upper) and quantified (bottom). The yellow arrow indicates the representative colocalization of RABV (red), TfR1 (green), and Rab5 (purple) (A), or Rab7 (purple) (B), shown in three dimensions. The Venn diagram represents the total amount of colocalization of RABV-TfR1 complex with Rab5 (A) or Rab7 (B). (C) Coimmunoprecipitation of TfR1-Flag with ERA G-Myc under acidic conditions (pH 5.5). The data shown are representative of three independent experiments.

We then investigated whether RABV is transported in the endosomes together with TfR1. Multiplex immunofluorescence with TSA staining was performed at 20 min after infection of N2a cells with ERA-N-mCherry. The result showed that TfR1 colocalized with ERA-N-mCherry in Rab5- or Rab7-positive endosomes in N2a cells (Fig. 8A and B). We also performed the coimmunoprecipitation assays under acidic conditions (pH 5.5) to detect the interaction between TfR1 and ERA G and found that the interaction is not affected by acidic conditions (Fig. 8C). These results indicate that RABV and TfR1 are cotransported to the early and late endosomes after infection.

## DISCUSSION

Our findings in this study strongly suggest that TfR1 is a novel host entry factor for RABV infection and that RABV directly interacts with TfR1 and hijacks the endocytosis of TfR1 to enter cells. It is somewhat surprising that RABV uses the endocytosis of TfR1 to enter cells. While both TfR1 and RABV can enter cells through CME (5, 6, 17), the lifetime of RABV endocytosis is much longer (>60 s) than that of classic TfR1 endocytosis and the process requires the assistance of actin (5, 28, 29), which differs from classic TfR1 endocytosis. Therefore, it is likely that other host factors that can delay the lifetime of TfR1 endocytosis are required for TfR1-mediated endocytosis of RABV. Interestingly, previous studies have showed that GPCR-containing clathrin-coated pits (CCPs) have longer cell surface residence times than TfR1-containing CCPs (30). The prolonged surface residence time is partially caused by tethering cargo to the actin cytoskeleton (30). Our previous study found that mGluR2, a member of GPCR family, is the entry receptor of RABV (13). In the future, it will be valuable to test whether mGluR2 or another GPCR family member mediates the endocytosis of RABV by interacting with TfR1.

It was reported that RABV is cotransported with Tf in rat primary neuronal cells (6). Our work extends this study. We found that RABV interacts and colocalizes with TfR1 in early and late endosomes, indicating that TfR1 is involved in RABV-containing endosomal transport. Under physiological conditions, TfR1 is transported to the early endosomes where iron is released from Tf, and the Tf-TfR1 complex recycles back to the plasma membrane (23, 31). A small proportion of the TfR1 is directed to the late endosomes for physiological turnover (32). The fusion of RABV mainly occurs in the late endosomes. It is therefore possible that RABV hijacks the signaling pathway that guides TfR1 to the late endosomes. Of note, our previous study found that mGluR2 colocalizes with RABV in early and late endosomes (13). It would be interesting to explore how TfR1 cooperates with mGluR2 to regulate the process of RABV endosomal transport in a future study.

The conclusions drawn from the present study are supported by the results obtained *in vitro* from siRNA silencing, protein interaction, antibody blocking, soluble protein neutralization, and image analysis. TfR1 is ubiquitously expressed in almost all tissues and the knockout of TfR1 is lethal under physiological conditions (17, 33). Therefore, we could not find or construct a TfR1-negative and RABV nonsusceptible cell line to test whether overexpression of TfR1 can render a cell line susceptible to RABV infection. For the same reason, we also could not construct TfR1 gene knockout mice to test the importance of TfR1 for RABV infection *in vivo*. In the future, it will be important to map the domain of TfR1 that interacts with RABV G, so that we could try to construct interacting domain-deletion mice to test the importance of TfR1 for RABV infection *in vivo*.

In conclusion, we found here that TfR1 is an entry factor of RABV infection. Given that TfR1 also is an important entry factor for multiple pathogens, including Machupo virus (21), canine parvovirus (34), and hepatitis C virus (35), targeting TfR1 may present a novel approach to develop pan-antiviral therapeutic interventions. Exploring the details of how TfR1 mediates RABV entry will help in pursuing this goal.

## MATERIALS AND METHODS

### Cell lines.

HEK293 cells (ATCC, CRL-1573), CHO cells (ATCC, CCL-61), and N2a cells (ATCC, CCL-131) were maintained in Dulbecco modified Eagle medium (DMEM) supplemented with 10% fetal bovine serum (FBS), 1% penicillin-streptomycin, and l-glutamine in 5% CO_2_. BSR-T7/5 cells were maintained in DMEM supplemented with 5% FBS, 1% penicillin-streptomycin, and l-glutamine at 37°C in 5% CO_2_.

### Primary neuron cell cultures.

Mouse primary neuron (mPN) cells were prepared by following previously established protocols (13). Briefly, newborn mice (within 3 days of birth) were sacrificed, the brain was removed, and the cortex was separated. Brain cortex was washed with PBS and cut into small pieces. Then, 0.25% trypsin was added to digest the cortex for 5 min at 37°C. DMEM supplemented with 10% FBS containing 100 μg/mL DNase I was added to the cells. The cells were filtered through a cell strainer and centrifuged at 1,000 rpm for 5 min. The cells were then cultured in neurobasal media (Thermo Fisher Scientific) supplemented with 10% B27 (Thermo Fisher Scientific), 2 mM glutamine, 25 mM HEPES, and 25 μg/mL β-d-arabinofuranoside. The cells were cultured for 4 to 6 days before use.

### Viruses.

RABV ERA strain and recombinant adenovirus 5 expressing EGFP (Ad-5-EGFP) were maintained in our laboratory (36, 37). Recombinant ERA expressing EGFP (ERA-EGFP) and recombinant ERA expressing mCherry, in which the ERA N and mCherry in-frame fusion gene was inserted between the ERA P and M genes as an additional transcription unit (ERA-N-mCherry), were generated as described previously (36, 38).

### Plasmids.

ERA G-Myc, GX/09 G-Myc, CVS-24 G-Myc, and WCBV G-Myc were described previously (13). Human TfR1 cDNA (NM_003234.4) and mouse TfR1 cDNA (NM_001357298.1) were cloned into the pCAGGS-Flag vectors as indicated in our study and confirmed by sequencing analysis.

### RNAi.

The RNAi assay was performed as previously described (26). Briefly, siRNA targeting the human TfR1 (1 μM, 10 μL per well; Ambion, s727), mouse TfR1 (5 μM, 4 μL per well; Dharmacon, L-055550-01-0005), TTP (1 μM, 10 μL per well; Ambion, s528796), RABV L (1 μM, 10 μL per well) or nontargeting siRNA, was mixed with Opti-MEM medium containing 1 μL of Lipofectamine RNAiMAX (Thermo Fisher Scientific, 13778150) transfection reagent in a volume of 120 μL per well on 24-well plates. After a 30-min incubation at room temperature, HEK293 cells (6 × 10^4^ cells/well) and N2a cells (5 × 10^4^ cells/well) were seeded into siRNA-coated 24-well plates in a volume of 500 μL per well. At 24 h after the siRNA transfection, TfR1 mRNA or TTP mRNA was assessed by use of qPCR. At 60 h posttransfection, the cells were infected with ERA-EGFP or Ad-5-EGFP for further studies. The siRNA sequences targeting RABV L were as follows: siRABV L, sense (5′-GGAAUGCACUUUCGAUAUAtt-3′) and antisense (5′-UAUAUCGAAAGUGCAUUCCtt-3′). The RNAi assay on 96-well plates was performed as previously described (13). At 60 h posttransfection, the cells were infected with ERA-EGFP at 37°C; the infection ratio of cells was determined at 48 h postinfection by using the Perkin-Elmer Operetta high-content system.

### Tf uptake assay.

At 60 h posttransfection, TfR1-silenced or TTP-silenced HEK293 cells or N2a cells were incubated with 200 μL of 50 μg/mL Alexa 488-labeled Tf (Thermo Fisher Scientific, T13342) in serum-free medium for 30 min at 37°C to allow Tf uptake. The cells were washed twice with cold PBS, then incubated for 2 min in cold stripping buffer (50 mM glycine, 100 mM NaCl [pH 3.0]) twice and then washed with cold PBS. After the surface bound Tf was removed, the cells were fixed with 3% paraformaldehyde and stained with Hoechst 33342 (Thermo Fisher Scientific, H3570). The fluorescence of the Alexa 488-labeled Tf was detected by using a Zeiss LSM880 laser-scanning confocal microscope.

### Viral infection assay.

For RNAi assays, at 60 h posttransfection, siRNA-transfected cells were infected with ERA-EGFP (multiplicity of infection [MOI] = 0.05) for 1 h at 37°C; for the overexpression assays, at 48 h posttransfection, the human TfR1-transfected CHO cells were infected with ERA-EGFP (MOI = 0.05) for 1 h at 37°C. The cells were then washed three times with 2% FBS-containing medium, and 2% FBS-containing medium was added. The supernatants were harvested at 24 and 48 h postinfection and titrated by serial dilution in BSR T7/5 cells. Viral titers are expressed as focus-forming units/mL.

To calculate the infection rate of ERA-EGFP, cells were fixed with 3% paraformaldehyde at 48 h postinfection, and the cell nuclei were stained with Hoechst 33342. The infection ratio was then determined by using the Perkin-Elmer Operetta high-content system.

### Flow cytometry.

At 60 h posttransfection, TfR1-silenced HEK293 cells were infected with Ad-5-EGFP (MOI = 0.1) or ERA-EGFP (MOI = 0.1) at 37°C for 1 h. The cells were washed three times with 2% FBS-containing medium and cultured with 2% FBS-containing medium. At 24 h postinfection, the cells were gently detached with 0.25% trypsin and washed twice with fluorescence-activated cell sorting (FACS) wash buffer (PBS containing 2% FCS) and then fixed with 3% paraformaldehyde at room temperature for 15 min. Cells were subsequently washed twice with FACS wash buffer prior to being analyzed in a FC500 flow cytometer (Beckman Coulter). The green fluorescence was measured and analyzed by using FlowJo software.

### FAC inhibition assay.

HEK293 cells (4 × 10^4^ cells/well), N2a cells (4 × 10^4^ cells/well), or mPN cells (3 × 10^4^ cells/well) were seeded onto 96-well plates. Cells were pretreated with FAC (Macklin, A800010) at the indicated concentrations for 1 h at 37°C. The cells were next infected with ERA-EGFP (MOI = 0.05) for 1 h at 37°C, thoroughly washed, and then cultured with 2% FBS-containing medium. FAC was present in the culture medium throughout the infection. At 48 h postinfection, the cells were fixed with 3% paraformaldehyde, and the cell nuclei were stained with Hoechst 33342. The infection ratio was then determined by using the Perkin-Elmer Operetta high-content system.

### Cell viability assay.

Cell viability was determined by using the Cell Titer Glo kit (Promega, G9242) according to the manufacturer’s instructions. HEK293 cells (4 × 10^4^ cells/well), N2a cells (4 × 10^4^ cells/well), or mPN cells (3 × 10^4^ cells/well) were seeded onto 96-well plates with opaque walls. Then, FAC or antibody at the indicated concentration was added; 48 h later, Cell Titer Glo reagent was added to each well. Luminescence was measured with a GloMax 96 microplate luminometer (Promega).

To detect the cell viability of TfR1- or TTP-silenced cells, HEK293 cells (6 × 10^4^ cells/well) and N2a cells (5 × 10^4^ cells/well) were seeded onto 24-well plates. Cell Titer Glo reagent was added to each well at 60 h posttransfection. The luminescence was measured as described above.

### Viral binding assay.

Cells were seeded onto 24-well plates and transfected with the indicated siRNA for 60 h or pretreated with MβCD (Sigma, C4555-1G) for 1 h. The cells were transferred onto ice for 20 min, the media were removed from the wells, and 200 μL of ERA-EGFP (MOI = 10) was added to the cells at 4°C for 1 h. After being washed with prechilled PBS three times to remove the unbound virions, the cells and bound virions were lysed by TRIzol (Thermo Fisher Scientific, 15596018) for qPCR to assess binding viral RNA.

### Viral entry assay.

Cells were seeded onto 24-well plates and transfected with the indicated siRNA for 60 h and then transferred onto ice for 20 min. The media were removed from the wells, and 200 μL of ERA-EGFP (MOI = 10) was added to the cells at 4°C for 1 h. After removal of unbound virions by extensive washing with chilled PBS, the cells were moved to 37°C for 2.5 h to allow the internalization of bound viruses. After 2.5 h, the cells were washed for 3 min with 0.25% trypsin to remove the cell surface-bound viruses, and the harvested cells were then lysed for total RNA extraction and subjected to qPCR to quantify entered viruses. The viral entry assay in MβCD-pretreated N2a cells was performed as described above, except virus entry was for 1 h at 37°C, and MβCD was present in the culture medium throughout the infection.

For microscopy, N2a cells were cultured on Millicell EZ slide 4-Well Glass (Merck Millipore, PEZGS0416) and transfected with the indicated siRNA for 60 h before the viral entry assay. After ERA-EGFP internalized at 37°C, the cells were immediately fixed with 3% paraformaldehyde for 15 min at room temperature. If needed, they were permeabilized with 0.1% Triton X-100 in PBS for 10 min at room temperature and then incubated with 1% BSA for 30 min to block nonspecific binding of antibodies. Both permeabilized and unpermeabilized cells were incubated with anti-RABV G mouse polyclonal antibody overnight at 4°C and then washed and stained with goat anti-mouse Alexa Fluor 488 (Thermo Fisher Scientific, A-11001) for 1 h. Nuclei were visualized by staining with Hoechst 33342. Fluorescence intensity was quantified with a Zeiss LSM880 laser-scanning confocal microscope (Carl Zeiss AG) equipped with Airyscan (Plan-Apochromat, objective 63×, 1.4 Numerical Aperture DIC oil immersion objective) using ZEN software. The cell-bound RABV signal intensities from at least 80 cells per sample were quantified using ZEN software.

### Real-time quantitative PCR.

To detect the level of TfR1 mRNA, TfR2 mRNA, TTP mRNA, and viral RNA in cells, total RNA from cells was isolated using TRIzol reagent; 2 μg of total RNA was used for reverse transcription (Vazyme). Relative mRNA expression was analyzed by using SYBR green qPCR Master Mix (Vazyme, Q711) with the indicated TfR1, TfR2, TTP, and RABV N primers. The 2^–ΔΔ^*^CT^* method was used to calculate the relative gene expression level, with β-actin as the internal control (39). Experiments were done in three biological replicates. The qPCR primers used were as follows: TfR1 (human), forward (5′-CAGCCCAGCAGAAGCATT-3′) and reverse (5′-CCAAGAACCGCTTTATCCAG-3′); TfR1 (mouse), forward (5′-AGGAACCAGACCGTTATGTTGTAG-3′) and reverse (5′-ACCTGTTCCCACACTGGACTTC-3′); TfR1 (Chinese hamster), forward (5′-GTGATTGTTAGAGCAGGG-3′) and reverse (5′-ACCAGTTCCTAGATGAGC-3′); TfR2 (mouse), forward (5′-GCCCAGAAGGTAGCCGTT-3′) and reverse (5′-GTCCGTACACAGCCTGGT-3′); TfR2 (Chinese hamster), forward (5′-TTCATCAGCTGGGACGGA-3′) and reverse (5′-TGTCCAGGCTCACGTACA-3′); TTP (human), forward (5′-ACGCACACAACACCACCGAAAT-3′) and reverse (5′-GCTACCTCGTCTGGGCTGTCTG-3′); β-actin (human), forward (5′-CGGGACCTGACTGACTACCTC-3′) and reverse (5′-CCATCTCTTGCTCGAAGTCCAG-3′); β-actin (mouse), forward (5′-CCTTCTTGGGTATGGAATCCTGTGG-3′) and reverse (5′-ACACAGAGTACTTGCGCTCAGGAGG-3′); β-actin (Chinese hamster), forward (5′-GTGCTGTCCCTGTATGCC-3′) and reverse (5′-AGAGCGTAGCCCTCATAG-3′); and RABV N, forward (5′-ATGAAGACTGTTCAGGACTGGTAT-3′) and reverse (5′-CCCTGGCTCAAACATTCTTCTTA-3′).

### Coimmunoprecipitation assay.

HEK293 cells (2 × 10^6^ cells/well) were seeded onto 6-well plates and transfected with plasmids by using TransIT-293 transfection reagent according to the manufacturer’s instructions. At 48 h posttransfection, the cells were lysed with the gentle extraction buffer, NP-40 lysis buffer (50 mM Tris [pH 7.4], 150 mM NaCl, 1% NP-40, 0.5% sodium deoxycholate), or the rigorous extraction buffer, radioimmunoprecipitation assay (RIPA) lysis buffer (50 mM Tris [pH 7.4], 150 mM NaCl, 1% Triton X-100, 1% sodium deoxycholate, 0.1% sodium dodecyl sulfate) for 1 h at 4°C. Supernatant was collected and mixed with 40 μL of protein G agarose (Roche, 11243233001) for 4 h at 4°C to remove nonspecific binding proteins in the supernatant. After being washed, the supernatant was mixed with anti-Flag antibody-conjugated agarose beads (Sigma, A2220) for 6 h at 4°C. The beads were isolated by centrifugation, washed five times with lysis buffer, and used for SDS-PAGE and Western blotting. The coimmunoprecipitation assay of ERA G and TfR1 under acidic conditions was performed as described above, except that the pH of the lysis buffer was adjusted to 5.5.

### Pulldown assay.

For pulldown assays, ERA G-His (amino acids [aa] 41 to 450) and TfR1-GST (aa 89 to 760) proteins were expressed in E. coli and purified by FriendBio Technology (Wuhan, Hubei, China). GST protein was used as the negative control. The purified GST-tagged proteins were respectively incubated with Glutathione Sepharose 4B beads (GE Healthcare Bioscience, 17-0756-01) at 4°C for 2 h. The beads were then washed and incubated with ERA G-His or whole-cell lysates from HEK293 cells expressing Flag-tagged or Myc-tagged proteins at 4°C for 5 h with constant rotation. After conjugation, the beads were washed five times with wash buffer (pH 8.5; 20 mM Tris, 500 mM NaCl, 2 mM EDTA) and resuspended in PBS and protein sample loading buffer. The samples were then subjected to SDS-PAGE and assessed by Western blotting.

### Western blotting.

Clarified cell lysate was diluted in denaturing SDS gel loading buffer, and boiled for 15 min. After denaturing, the samples were loaded onto a 4 to 12% gel (GenScript, M41210C) for SDS-PAGE and separated by electrophoresis. Proteins were transferred to a polyvinylidene difluoride (PVDF) membrane (Merck-Millipore, ISEQ00010). The PVDF membrane was blocked with 5% skim milk in PBS containing 0.1% Tween 20, and then incubated with the following primary antibodies: anti-Flag antibody (GenScript, A00187), anti-Myc antibody (GenScript, A00172), anti-GST antibody (GenScript, A00097), or anti-His antibody (GenScript, A00174). The PVDF membrane was then washed three times with PBS and incubated with horseradish peroxidase (HRP)-conjugated goat anti-mouse antibody (GenScript, A00160) and goat anti-rabbit antibody (GenScript, A00098). After three washes with PBST buffer, target protein bands were detected by using the enhanced chemiluminescence (ECL) reagent (Merck Millipore, WBLUR0500).

### Antibody blocking assay.

HEK293 cells (4 × 10^4^ cells/well), N2a cells (4 × 10^4^ cells/well), or mPN cells (3 × 10^4^ cells/well) were seeded onto 96-well cell carrier plates (Perkin-Elmer, 6055302). The media were removed from the well, and the cells were treated with the indicated concentrations of TfR1 antibody (BD Pharmingen, 555534), or isotype antibody IgG2a (20 μg/mL; Southern Biotech, 0103-01) for 1 h on ice. Cells were then incubated with ERA-EGFP (MOI = 0.05) for 1 h at 4°C in the presence of the indicated concentrations of antibody. They were then washed and incubated with medium containing the indicated antibodies at 37°C. At 48 h postinfection, the cells were fixed with 3% paraformaldehyde, and the nuclei were stained with Hoechst 33342. The infection ratio was determined by using the Perkin-Elmer Operetta high-content system.

### Soluble TfR1 ectodomain neutralization assay.

HEK293 cells (4 × 10^4^ cells/well), N2a cells (4 × 10^4^ cells/well), or mPN cells (3 × 10^4^ cells/well) were seeded onto 96-well carrier plates. ERA-EGFP (MOI = 0.05) was mixed with the purified soluble TfR1-GST protein at the indicated concentrations or with GST at 4°C for 1 h. The media were removed from the well, and the cells were incubated with the virus-protein mixture at 37°C for 1 h. The cells were then washed and incubated with growth medium. At 48 h postinfection, the cells were fixed with 3% paraformaldehyde, and the nuclei were stained with Hoechst 33342. The infection ratio was determined by using the Perkin-Elmer Operetta high-content system.

### Multiplex immunofluorescence.

N2a cells (5 × 10^4^ cells/well) were cultured on Millicell EZ slide 4-Well Glass and then infected with ERA-N-mCherry (MOI = 10) at 37°C for 10 min or 20 min. The cells were thoroughly washed with PBS and fixed with 3% paraformaldehyde. Multiplex immunofluorescence with tyramide signal amplification (TSA) was performed by following the previously established protocol (26). Briefly, endogenous peroxidase activity was quenched. After permeabilization with 0.1% Triton X-100 and blocking steps (Zsbio, ZLI-9056), samples were incubated with primary antibodies followed by HRP-conjugated secondary antibodies. Multiplex fluorescence labeling was performed using TSA-Dendron-fluorophores (NEON 7-color Allround Discovery kit for FFPE; Histova Biotechnology, NEFP750). A commercial antibody stripping buffer was employed to remove the primary and secondary antibodies while retaining the TSA signal by incubation for 30 min at 37°C. After a brief rinse, other antigens were serially detected by using spectrally different TSA reagents and following the above method. The primary antibodies used in this study were: TfR1 antibody (Santa Cruz, sc-32272), Rab5 antibody (Abcam, ab13253), Rab7 antibody (Abcam, ab137029), and mCherry antibody (Abcam, ab183628). The secondary antibodies were HRP-conjugated anti-rabbit IgG antibody (Zsbio, PV-6001) and HRP-conjugated anti-mouse IgG antibody (Zsbio, PV-6002). Images were acquired by using a Zeiss LSM880 laser-scanning confocal microscope equipped with Airyscan. The resolution of the acquired image was 1,024 × 1,024.

### Statistical analysis.

Quantitative data are presented as means ± the standard deviations (SD) of three independent experiments or replicates. The statistical analysis of the normalized data was performed using Microsoft Excel with an unpaired two-tailed Student *t* test. The statistical details are given in the respective figure legends. Significance levels are indicated in the figures as follows: ns, not significant; *, *P* < 0.05; **, *P* < 0.01; and ***, *P* < 0.001.

RABV-infected cell images were acquired by using the Perkin-Elmer Operetta high-content system (13). At least 40 fields per well were acquired at 20 × magnification. Columbus software (Perkin-Elmer) was used to automatically identify and quantify the green fluorescence-positive areas and cell nuclei. The infection ratio was expressed as the quotient of the total cell numbers divided by the infected cell numbers per well.

The colocalization analysis was performed by using Bitplane Imaris software. Imaris software was used for three-dimensional reconstruction, colocalization, and statistical analysis. For the colocalization analysis of RABV and TfR1, the channels were processed by using “surface module.” The surface results of the red channel (RABV) and green channel (TfR1) were processed by using the “spot module,” and then the respective spots were counted. The surface results of RABV and TfR1 were merge into one channel by using the “coloc module.” The new channel was processed by using the “spot module.” The spots from the new channel were then counted.

Colocalization analysis of RABV, TfR1, and Rab5 or Rab7 was performed as previously described (13).

## References

[1] Davis BM, Rall GF, Schnell MJ. 2015. Everything you always wanted to know about rabies virus (but were afraid to ask). Annu Rev Virol 2:451–471. 10.1146/annurev-virology-100114-055157. 26958924 PMC6842493

[2] Gaudin Y, Ruigrok RW, Tuffereau C, Knossow M, Flamand A. 1992. Rabies virus glycoprotein is a trimer. Virology 187:627–632. 10.1016/0042-6822(92)90465-2. 1546457 PMC7131270

[3] Etessami R, Conzelmann KK, Fadai-Ghotbi B, Natelson B, Tsiang H, Ceccaldi PE. 2000. Spread and pathogenic characteristics of a G-deficient rabies virus recombinant: an *in vitro* and *in vivo* study. J Gen Virol 81:2147–2153. 10.1099/0022-1317-81-9-2147. 10950970

[4] Schnell MJ, McGettigan JP, Wirblich C, Papaneri A. 2010. The cell biology of rabies virus: using stealth to reach the brain. Nat Rev Microbiol 8:51–61. 10.1038/nrmicro2260. 19946287

[5] Piccinotti S, Kirchhausen T, Whelan SP. 2013. Uptake of rabies virus into epithelial cells by clathrin-mediated endocytosis depends upon actin. J Virol 87:11637–11647. 10.1128/JVI.01648-13. 23966407 PMC3807345

[6] Piccinotti S, Whelan SP. 2016. Rabies internalizes into primary peripheral neurons via clathrin-coated pits and requires fusion at the cell body. PLoS Pathog 12:e1005753. 10.1371/journal.ppat.1005753. 27463226 PMC4963122

[7] Maginnis MS. 2018. Virus-receptor interactions: the key to cellular invasion. J Mol Biol 430:2590–2611. 10.1016/j.jmb.2018.06.024. 29924965 PMC6083867

[8] Grove J, Marsh M. 2011. The cell biology of receptor-mediated virus entry. J Cell Biol 195:1071–1082. 10.1083/jcb.201108131. 22123832 PMC3246895

[9] Lafon M. 2005. Rabies virus receptors. J Neurovirol 11:82–87. 10.1080/13550280590900427. 15804965

[10] Lentz TL, Burrage TG, Smith AL, Tignor GH. 1983. The acetylcholine receptor as a cellular receptor for rabies virus. Yale J Biol Med 56:315–322. 6367238 PMC2589619

[11] Thoulouze MI, Lafage M, Schachner M, Hartmann U, Cremer H, Lafon M. 1998. The neural cell adhesion molecule is a receptor for rabies virus. J Virol 72:7181–7190. 10.1128/JVI.72.9.7181-7190.1998. 9696812 PMC109940

[12] Tuffereau C, Benejean J, Blondel D, Kieffer B, Flamand A. 1998. Low-affinity nerve-growth factor receptor (P75NTR) can serve as a receptor for rabies virus. EMBO J 17:7250–7259. 10.1093/emboj/17.24.7250. 9857182 PMC1171071

[13] Wang J, Wang Z, Liu R, Shuai L, Wang X, Luo J, Wang C, Chen W, Wang X, Ge J, He X, Wen Z, Bu Z. 2018. Metabotropic glutamate receptor subtype 2 is a cellular receptor for rabies virus. PLoS Pathog 14:e1007189. 10.1371/journal.ppat.1007189. 30028877 PMC6070288

[14] Shuai L, Wang J, Zhao D, Wen Z, Ge J, He X, Wang X, Bu Z. 2020. Integrin β1 promotes peripheral entry by rabies virus. J Virol 94:e01819-19. 10.1128/JVI.01819-19. 31666383 PMC6955245

[15] Wang C, Wang J, Shuai L, Ma X, Zhang H, Liu R, Chen W, Wang X, Ge J, Wen Z, Bu Z. 2019. The serine/threonine kinase AP2-associated kinase 1 plays an important role in rabies virus entry. Viruses 12. 10.3390/v12010045. PMC7019586 31905947

[16] Luo J, Zhang Y, Wang Y, Liu Q, Chen L, Zhang B, Luo Y, Huang S, Guo X. 2020. Rhabdovirus infection is dependent on serine/threonine kinase AP2-associated kinase 1. Life (Basel) 10. 10.3390/life10090170. PMC7554979 32872567

[17] Gammella E, Buratti P, Cairo G, Recalcati S. 2017. The transferrin receptor: the cellular iron gate. Metallomics 9:1367–1375. 10.1039/c7mt00143f. 28671201

[18] Kawabata H. 2019. Transferrin and transferrin receptors update. Free Radic Biol Med 133:46–54. 10.1016/j.freeradbiomed.2018.06.037. 29969719

[19] Kawabata H, Yang R, Hirama T, Vuong PT, Kawano S, Gombart AF, Koeffler HP. 1999. Molecular cloning of transferrin receptor 2: a new member of the transferrin receptor-like family. J Biol Chem 274:20826–20832. 10.1074/jbc.274.30.20826. 10409623

[20] Ward JH, Kushner JP, Kaplan J. 1982. Regulation of HeLa cell transferrin receptors. J Biol Chem 257:10317–10323. 10.1016/S0021-9258(18)34022-5. 6286649

[21] Radoshitzky SR, Abraham J, Spiropoulou CF, Kuhn JH, Nguyen D, Li W, Nagel J, Schmidt PJ, Nunberg JH, Andrews NC, Farzan M, Choe H. 2007. Transferrin receptor 1 is a cellular receptor for New World haemorrhagic fever arenaviruses. Nature 446:92–96. 10.1038/nature05539. 17287727 PMC3197705

[22] Uhlen M, Fagerberg L, Hallstrom BM, Lindskog C, Oksvold P, Mardinoglu A, Sivertsson A, Kampf C, Sjostedt E, Asplund A, Olsson I, Edlund K, Lundberg E, Navani S, Szigyarto CA, Odeberg J, Djureinovic D, Takanen JO, Hober S, Alm T, Edqvist PH, Berling H, Tegel H, Mulder J, Rockberg J, Nilsson P, Schwenk JM, Hamsten M, von Feilitzen K, Forsberg M, Persson L, Johansson F, Zwahlen M, von Heijne G, Nielsen J, Ponten F. 2015. Proteomics: tissue-based map of the human proteome. Science 347:1260419. 10.1126/science.1260419. 25613900

[23] Hsu VW, Bai M, Li J. 2012. Getting active: protein sorting in endocytic recycling. Nat Rev Mol Cell Biol 13:323–328. 10.1038/nrm3332. 22498832

[24] Rodal SK, Skretting G, Garred O, Vilhardt F, van Deurs B, Sandvig K. 1999. Extraction of cholesterol with methyl-beta-cyclodextrin perturbs formation of clathrin-coated endocytic vesicles. Mol Biol Cell 10:961–974. 10.1091/mbc.10.4.961. 10198050 PMC25220

[25] Subtil A, Gaidarov I, Kobylarz K, Lampson MA, Keen JH, McGraw TE. 1999. Acute cholesterol depletion inhibits clathrin-coated pit budding. Proc Natl Acad Sci USA 96:6775–6780. 10.1073/pnas.96.12.6775. 10359788 PMC21991

[26] Wang J, Yang G, Wang X, Wen Z, Shuai L, Luo J, Wang C, Sun Z, Liu R, Ge J, He X, Hua R, Wang X, Yang X, Chen W, Zhong G, Bu Z. 2021. SARS-CoV-2 uses metabotropic glutamate receptor subtype 2 as an internalization factor to infect cells. Cell Discov 7:119. 10.1038/s41421-021-00357-z. 34903715 PMC8668938

[27] Tosoni D, Puri C, Confalonieri S, Salcini AE, De Camilli P, Tacchetti C, Di Fiore PP. 2005. TTP specifically regulates the internalization of the transferrin receptor. Cell 123:875–888. 10.1016/j.cell.2005.10.021. 16325581

[28] Cureton DK, Massol RH, Whelan SP, Kirchhausen T. 2010. The length of vesicular stomatitis virus particles dictates a need for actin assembly during clathrin-dependent endocytosis. PLoS Pathog 6:e1001127. 10.1371/journal.ppat.1001127. 20941355 PMC2947997

[29] Cureton DK, Massol RH, Saffarian S, Kirchhausen TL, Whelan SP. 2009. Vesicular stomatitis virus enters cells through vesicles incompletely coated with clathrin that depend upon actin for internalization. PLoS Pathog 5:e1000394. 10.1371/journal.ppat.1000394. 19390604 PMC2667253

[30] Puthenveedu MA, von Zastrow M. 2006. Cargo regulates clathrin-coated pit dynamics. Cell 127:113–124. 10.1016/j.cell.2006.08.035. 17018281

[31] Tachiyama R, Ishikawa D, Matsumoto M, Nakayama KI, Yoshimori T, Yokota S, Himeno M, Tanaka Y, Fujita H. 2011. Proteome of ubiquitin/MVB pathway: possible involvement of iron-induced ubiquitylation of transferrin receptor in lysosomal degradation. Genes Cells 16:448–466. 10.1111/j.1365-2443.2011.01499.x. 21392187

[32] Fujita H, Iwabu Y, Tokunaga K, Tanaka Y. 2013. Membrane-associated RING-CH (MARCH) 8 mediates the ubiquitination and lysosomal degradation of the transferrin receptor. J Cell Sci 126:2798–2809. 10.1242/jcs.119909. 23606747

[33] Ding H, Chen S, Pan X, Dai X, Pan G, Li Z, Mai X, Tian Y, Zhang S, Liu B, Cao G, Yao Z, Yao X, Gao L, Yang L, Chen X, Sun J, Chen H, Han M, Yin Y, Xu G, Li H, Wu W, Chen Z, Lin J, Xiang L, Hu J, Lu Y, Zhu X, Xie L. 2021. Transferrin receptor 1 ablation in satellite cells impedes skeletal muscle regeneration through activation of ferroptosis. J Cachexia Sarcopenia Muscle 12:746–768. 10.1002/jcsm.12700. 33955709 PMC8200440

[34] Goodman LB, Lyi SM, Johnson NC, Cifuente JO, Hafenstein SL, Parrish CR. 2010. Binding site on the transferrin receptor for the parvovirus capsid and effects of altered affinity on cell uptake and infection. J Virol 84:4969–4978. 10.1128/JVI.02623-09. 20200243 PMC2863798

[35] Martin DN, Uprichard SL. 2013. Identification of transferrin receptor 1 as a hepatitis C virus entry factor. Proc Natl Acad Sci USA 110:10777–10782. 10.1073/pnas.1301764110. 23754414 PMC3696786

[36] Shuai L, Feng N, Wang X, Ge J, Wen Z, Chen W, Qin L, Xia X, Bu Z. 2015. Genetically modified rabies virus ERA strain is safe and induces long-lasting protective immune response in dogs after oral vaccination. Antiviral Res 121:9–15. 10.1016/j.antiviral.2015.06.011. 26093157

[37] Wang J, Luo J, Wen Z, Wang X, Shuai L, Zhong G, Wang C, Sun Z, Chen W, Ge J, Liu R, Wang X, Bu Z. 2022. Alpha-soluble NSF attachment protein prevents the cleavage of the SARS-CoV-2 Spike protein by functioning as an interferon-upregulated furin inhibitor. mBio 13:e02443-21. 10.1128/mbio.02443-21. PMC8749436 35012335

[38] Zhao D-D, Shuai L, Ge J-Y, Wang J-L, Wen Z-Y, Liu R-Q, Wang C, Wang X-J, Bu Z-G. 2019. Generation of recombinant rabies virus ERA strain applied to virus tracking in cell infection. J Integrative Agriculture 18:2361–2368. 10.1016/S2095-3119(19)62717-6.

[39] Wang X, Luo J, Wen Z, Shuai L, Wang C, Zhong G, He X, Cao H, Liu R, Ge J, Hua R, Sun Z, Wang X, Wang J, Bu Z. 2022. Diltiazem inhibits SARS-CoV-2 cell attachment and internalization and decreases the viral infection in mouse lung. PLoS Pathog 18:e1010343. 10.1371/journal.ppat.1010343. 35176124 PMC8890723

